# The Role of Teachers’ Grit and Motivation in Self-Directed Professional Development

**DOI:** 10.3389/fpsyg.2022.922693

**Published:** 2022-06-16

**Authors:** Yuling Lan

**Affiliations:** Department of College English, Qiqihar University, Qiqihar, China

**Keywords:** grit, motivation, self-directed professional development, EFL teachers, English language teaching

## Abstract

Through English language teaching (ELT), educator professional development is an interesting and significant subject due to the fact it is in the interest of the educators’ career to make sure that its participants perform consistently with the highest degrees of agreed criteria. Correspondingly, educators mostly state a willingness to enhance their own education that allows them to enhance learners’ knowledge and consistent with the advent of the information-based community, the self-directed learning is vital for the employees. The self-directed professional development (PD) method is considered one option to improve vocational educators’ skills to improve their professionalism, learning development, and school development. Within the learning procedure, self-directed PD has used the rules of learning that are covered within the rules of individual teaching and the mental traits and ideas including motivation and grit that could have a mediating function in this area. Grit is critical within English language educator PD and improving it within educator PD means modeling it as a determinant of the learners’ success. In addition, comprehending motivation is vital for everybody intending to reduce expenses for PD, motivate employees, or be self-directed in their learning about the job. The purpose of the present literature review is to examine the function of such constructs in self-directed PD which is considered a great dimension of educator quality. Briefly, many applications are suggested for the scholastic beneficiaries.

## Introduction

Conventionally, education has been considered as the conveying of information from one head to another and the education performance transmits knowledge and encourages learners, increases learners’ self-assurance, grows self-assurance, encourages, and constructs a positive learning setting (Muijs et al., 2005). Recently, in studies on educators and educator development and growth, the PD concept is taken into account, developing research on educator training, growth, development, and educator growth tactics (Ravandpour, 2019). Educators’ PD is the component of any academic organization coping with the readiness and education of educators to attain the needed competencies and skills in education for improving the educators’ quality in their courses, and educators’ PD consists of tasks that are studied by educators when they finish their educator training workshops (Shawer, 2010). Nowadays, educators have a high level of education that always enhances competencies, and accumulate new skills by getting engaged in lifelong learning. They are constantly engaged in their PD alongside their career. Simultaneously, enhancing expectancies from educator trainers by governmental institutes obligate the community to work hard, which intensifies the difficulties in different ways such as method, material, competencies, and manner (Thakur, 2012). To address these demanding situations, educators ought to possess a sense of motivation for continual and career-long learning, which expediates sustained, mental, and career-centered adulthood (Derakhshan, 2022). For the profession to keep up with changes, educators renew and revise their competencies, information, viewpoint, and conduct. They are engaged in multiple learning tasks like self-directed learning, cooperative learning, thoughtful activities, and digital learning, which assist them to achieve PD (Bhatt, 2021). A critical element for a prosperous search for work alludes to PD, which is described as tasks designed to assist learners to get ready for succeeding in the university-work transition (Blau et al., 2014). Learning opportunity educators participate to enhance their knowledge and to continue to be at the cutting-edge of effective education tactics and strategies, and this approach is also part of PD (Alibakshi and Dehvari, 2015). Educator PD is beyond the sequence of education workshops, institutions, conferences, and daily in-service. It is a learning procedure of how to make knowledge practical by engaging in practice inside a practitioners’ network (Schlager and Fusco, 2003). Educator PD across an individual’s profession has an aspect of self-directed PD in terms of the educator taking the initiative and possessing the determination to learn (Zepeda, 2013).

Self-directed PD is a field wherein the educators or trainers evaluate themselves and emphasize the fields wherein they require enhancements and guidance and teach themselves to address those difficulties. The roles, structures, and performance of the academic institutes are modified and modifications are regularly held so the duties and tasks of the educators are converted simultaneously. This is educators’ obligation to teach their learners to fulfill the requirements of the new instructional systems such as new evaluation measures, principles, exams, etc. (Djatmiko, 2011).

Within a self-directed PD, the educators and the professionals evaluate their vulnerable fields and emphasize them and enhance them with their tactical speed. It paves the way for the lecturers or the teachers to broaden their educating and learning competencies with a genuine source at any location and time (Lopes and Cunha, 2017). Self-directed PD engages the educators in tasks which improve their career competencies and make new tactics practical. Teachers’ control over their experiences and duties are assigned to them to find new answers to the difficulties (Morris, 2019). The procedure of self-directed PD refers to learning taken and originated by oneself. In such a procedure, the students evaluate their weaknesses and enhance their educational approaches and delivery speeds (Djatmiko, 2011). It is contended that even though educators’ dynamic and group involvement in PD is crucial, the depth, significance, and relevance of the novel information as well as educators’ skill to approach and employ novel information instantly in their classes is important to the enterprise of educator learning.

A feature of people affecting educators’ implementation is educators’ motivation to execute (Kennedy, 2016). For example, scholars have linked educators’ choice to use their education, their understood worth of executing, and the extent to which they consider their success in execution when executing consequences, namely altered education activities (Emo, 2015; Gaines et al., 2019). Having higher particularity for an academic environment, motivation takes place when people feel a feeling of autonomy, experience a link to the school, and experience that they have the needed competence vital for succeeding in a school (Ryan and Deci, 2020). Educators’ motivation has an important function in specifying what takes place after educators take part in PD. Educators’ execution after PD is a roughly specific environment, and it is essential to be aware that the enriched learning essential for educators to execute new activities is often not obtained through the time spent in formal PD classes (Kyndt et al., 2014). Moreover, by placing educators’ motivation to learn at the crossing point, scholars are encouraged to dig more profoundly and go beyond the present perceptions of PD to integrate educators’ motivation to learn in an attempt to develop a deeper theoretical and practice-based comprehension of educators’ professional learning.

Another notion in this field is grit, because all people have grit or determination to various degrees, and grit is highly beneficial for people in many life situations (Wang et al., 2021; Widodo, 2021). Grit is a method of determining whether or not a person can manage to survive when encountering difficulties in life (Hochanadel and Finamore, 2015) and mirrors a mental factor that gave importance to perseverance as a predictor of long-run achievement and is related to attaining high-level objectives for a long duration (Von Culin et al., 2014; Duckworth, 2016). For instance, grit forecasts essential living results, describing a unique, though minor, variation in educational achievement or career retention (Eskreis-Winkler et al., 2014). Grit also pertains to compatible results among younger and adult people, like job satisfaction, job functioning, effective results and motivation (Credé et al., 2017; Guo et al., 2019), and student educational success. Moreover, quantifying grit could predict the related university and graduate faculty GPA and success (Duckworth, 2016). Grit pertains to awareness and self-management, with people who indicate high degrees of grit showing the perseverance to complete apparently impossible activities. People with higher degrees of grit often experience higher levels of success in different fields of their life (Credé et al., 2017).

With the development of the literature on educator PD, researchers have started to regard the proper measures for educators as mature students (Smith, 2017). This indicates a considerable change within the core of PD scholars, who were more conscious of the crucial role educators have in their learning. As SDL depends on the student, when it is taken by educators, they turn into the incentive behind their professional learning, i.e., they become conscious about their accountability in respect to figuring out the fields that require development, and work independently to deal with these fields. In addition, institutes put great preference on the professionalization of educators’ which they use. One function of educator PD is to present a condition through which educators can keep developing and help in retaining the educators’ attraction, innovation, and passion in their career (Richards, 2015).

Also in the literature review, it is stated that educators’ engagement in self-directed learning mostly relies upon the mixture of educators’ internal motivation to learn, their passion and perseverance, and organizational help that clarifies that educators may get the strength to manage their learning experiences (Lopes and Cunha, 2017; Louws et al., 2017). Due to the above-mentioned issue, the researcher makes an effort to review the literature on the role of teachers’ factors such as grit and motivation in the process of self-directed PD.

## Review of the Literature

### Motivation

Self-Determination Theory (SDT) is related to people’s motivation and the place where motivation is evolved: from inside one’s self or from an extrinsic source. Moreover, SDT scholars try to distinguish if people’s motivation originates from their choices whether it comes from them being driven by their goals and desires (Wehmeyer et al., 2016; Koole et al., 2019). Self-Determination Theory pertains to peoples’ motivation, health, and growth (Deci and Ryan, 2016) and it is a complete theory that includes multiple smaller theories. Basic Needs Theory is one of those theories, stating that human beings effort to satisfy three inner desires, namely, autonomy, relatedness, and skill (Hagger et al., 2006). Autonomy alludes to the requirement for feeling in control of one’s behaviors and self-directed goals. That is, if human beings sense that they can take measures leading to an alteration, they may become self-determined humans (Derakhshan et al., 2020). Respectively, it is found that because the educators in the study were free to select the substances they needed within the class, their motivation was at a high level (Bradley, 2010). Relatedness is involved with the attachment and belonging of humans to others. Individuals try to be engaged in a social group and sense a relationship with them. Ultimately, skill alludes to the desire to indicate one’s level of command in specific competencies, and assignments are identified by others because of one’s knowledge of those assignments. Educators’ motivation for pursuing PD is driven by numerous sources and falls into “extrinsic” and “internal” motivations. There is the extrinsic requirement for the industrial relationships frame to adopt PD or the internal motivation to perform a career well (Henderson, 2012).

Motivation provides the main driving force in language learning, and after a while, it is deemed as the reason for seeking the expanded and tedious academic process (Dörnyei and Ryan, 2015). They believed that motivation contributes to the whole elements connected to gaining knowledge of a second language. Furthermore, it is stated that motivation is associated with the justification behind people’s specific decision-making, participation in a task, and persistence in following it and it adjusts the magnitude of strengths and personal participation in learning a second language (Ushioda, 2013). Motivation can be defined as preparing and aspiring to take part in good teaching. There are two different types of motivation: independent and controlled motivation. While independent motivation alludes to behaving with a sense of resolution and dealing with options, controlled motivation refers to behaving with a sense of force (Fokkens-Bruinsma et al., 2018). Independent motivation is connected to higher levels of psychological health, more determination and purposefulness, higher cognitive skills, higher levels of work fulfillment, and organizational commitment (Fokkens-Bruinsma et al., 2018).

### Grit

Recently, Duckworth (2016) developed the Grit theory which links the constructs of enthusiasm and persistence to peoples’ potential to effectively reach their goals. Grit indicates the reason some humans succeed in pursuing their goals and the reason some humans fail, which is described as “enthusiasm and persistence for long-run objectives” (Duckworth et al., 2007). Grit is the power people have to continue pursuing their long-term life objectives regardless of experiencing challenges, disasters, or failures (Duckworth and Gross, 2014; Robertson-Kraft and Duckworth, 2014; Duckworth, 2016). Grittier people consider life as a marathon and indicate a high work dedication. The grit construct does not predispose people not to experience difficulties and problems, however, they can keep concentrated and move towards their final purpose (Dale et al., 2018). Grit has been demonstrated to be a possible motivator in presentation (Von Culin et al., 2014) and indicative of people’s attendance and retention in the areas of military service, career, marriage, and high school (Eskreis-Winkler et al., 2014).

Duckworth (2016) divided grit into the two elements of enthusiasm and persistence and every element has a collective as well as an independent effect on people’s capability to develop and maintain grit. Enthusiasm is a powerful love or desire for a specific task or notion. Enthusiasm can begin by being intrinsically performed through involvement in the task wherein the criteria are met. The interest consistency is maintained by the goal that it presents, both individually and professionally. Consistency is a factor of the trait-level grit that directs people in dedicating themselves to targeted, sincere, and exceedingly difficult competence activities that end in learning. Consistency lets people let go of instantaneous and intermediate preferences and goals for long-term success (Duckworth, 2016).

Grit is developed over a lifetime of learning to address and overcome refusal and failure (Duckworth, 2016). Grit is fostered when people realize the distinction between low-order and high order objectives and learn where to direct their energies. According to Duckworth, talent does not make people gritty, however, the tendency to preserve learning and development through one’s enthusiasm for a task. Grit includes two indices, first, perseverance of pursuits, which indicates people’s willingness to remain dedicated and concentrated on attaining objectives/duties over a long duration; and perseverance of effort reflects people’s willingness to pursue long-run targets with continued attempts regardless of limitations and problems (Duckworth and Quinn, 2009). If such indices are sufficient and persistent in the long term, we may expect an enhancement of professional skills.

### Self-Directed Professional Development

Professional development is a non-stop effort that is specific to the context, which is directed by standards, embedded inside the educators’ task, centered on learners’ learning, and suited to educators’ levels of career growth (Schlager and Fusco, 2003). It also pertains to educators’ learning and conveying their information into action for the sake of learners’ growth (Avalos, 2011). Professional development is developed to improve tasks, and the procedure of PD is improved in the best way through the compliance with educators’ objectives, district requirements, and learner evaluations, regardless of it is for individual or societal learning, or forced upon educators (Colbert et al., 2008). Through PD, individuals retain the knowledge and capabilities around their work-life (Collin et al., 2012) which could manifest in different shapes, from formal instructional classes to learning through daily work activities; however, in its most easily identified shape, it refers to improving professional knowledge through brief formal courses presented through working teams (Collin et al., 2012). PD aims at increasing educators’ quality because educator quality is a vital component of learning (Geringer, 2003). As educators learn, they instinctively become cooperative, thoughtful, and interested in methods of solving problems facing education and learning (Mitchell and Sackney, 2019).

Self-directed PD refers to the PD originating from the educators’ own initiative, that is, the procedure is internally specified and started (Mushayikwa and Lubben, 2009). Contrary to PD given or guided by the school structure or area director, the educator specifies the timeline and peers taking part in this type of PD. Self-directed PD refers to learning initiated by oneself and expands professionally using self-motivation, initiative, and powerful determination (Zepeda, 2013). Educators initiate their PD, which has higher effectiveness than other instructions, and Self-directed PD improves the experience of self-image and self-assessment that lays the groundwork for improvement. It improves educators’ self-concept, where educators can handle their professional experiences and are motivated by assignments or problems that are meaningful to them (Bhatt, 2021).

Self-directed PD refers to the continuing and constant procedure where enhancements in education and learning arise step by step through the professional teaching of educators (Saleem and Shahid, 2021). In addition, self-directed PD consists of the adjustments and acceptance of these modifications by teachers and learners in the following methods: efficient comprehension, efficient education, educational material, flourishing the students, improvement of the professionalism feeling and information of the language, society, and the various cultures. In self-directed PD, a learner became responsible improving their learning to alter their belief in the direction of novel technologies and tactics (Shurr et al., 2014). Educators who are included in self-directed professional growth, self-control their education and deal with their societal and contextual settings to attain and affect their educational objectives (Buzza and Allinotte, 2013; Cho and Heron, 2015). Through self-control, educators oversee their educational demands and go after educational chances to satisfy their demands. Through self-control, educators’ convictions lead them in aligning their professional educational demands as they advance in their teaching profession as lifelong students (Spruce and Bol, 2015).

## Conclusion

The programs of self-directed PD are the cornerstone for lecturers should their PD recognize the demand for enhancements and modern information. Educators employ such developmental programs for their job progression and career consent. Such programs are conducted in academic environments as well for changing the conventional academic tactics and improving and applying modern information to learners. In self-directed types of PD, the educator is held accountable to determine several PD objectives and select the suitable forms of actions that will assist him or her with achieving these objectives (Villegas-Reimers, 2003). Self-directed PD offered educator-subjects independent, strengthening encounters as they made professional judgments about the time, setting, and material of their professional educational encounters.

Within the perspective of the theory of self-determination, three fundamental requirements ought to be met for health and development to happen: (a) competence, (b) relatedness, and (c) autonomy (Orsini et al., 2015). Motivation can grow whilst these primary requirements are fully satisfied, and declines when these three fundamentals are not satisfied (Ryan and Deci, 2020). In both scenarios, SDT has main applications pertaining to motivation that educators retain regarding the possibility of participating in self-directed PD. Competence in this context alludes to the subjects’ capacity to learn their education exercises, that they are fulfilling things within the eyes of learners and co-workers, and it appears common that on the whole educators desired to perform perfectly for their learners. In the same vein, there was a great help from introjected, biased, external motivation regarding the necessity to be regarded as a successful and skilled colleague in a school, specifically among inexperienced educators. They must be prompted to employ PD as a way to enhance competence. Also, relatedness pertains to educators sharing a confident relationship both with educator colleagues and head colleagues. Similar to the advantages of a healthy culture in the school, a healthy relationship between the educator and head is essential in flourishing whatever motivation educators need to involve in PD. Autonomy permits people to possess strength of decision-making concerning the PD options accessible to them and it considers individual priorities and judgments. In the PD domain, autonomy became vital for all individuals considering the motivation to follow PD. Because the focus is on self-directed PD, there have been several inner tendencies on the shoulder of all members to hold on to such PD, as self-direction and autonomy are identical theoretically. When educators’ autonomy declines, their motivation to involve in PD also declines; therefore, maintaining autonomy in PD chances that may emerge within the academic year is significant.

Since self-directed PD is an autonomous learning method that makes educators more responsible and is less reliant upon the educational establishment, it needs the perseverance and effort of teachers and it calls for educators to be independent to fulfill the particular requirements of their class as they continuously involve in self-motivated and self-directed learning. In this process, motivation has an important function in attaining novel information, novel abilities, novel strategies, novel comprehension, etc. Undoubtedly, it is an innate force to carry out an action and assists with achieving the objective.

Motivation is required for self-directed PD. Internal motivation inspires people to have more concern and dynamism, which aids in being thoughtful, conscious, and up to date. Self-direction is the present requirement to make adequate and proper modifications in education and learning. The self-directed learning PD method improves the teaching quality and tremendously affects development (Soebari and Aldridge, 2015). Self-directed PD assists educators in catching up with the growing quantity of knowledge relative to their career, copes with peoples’ needs, and inspires a high degree of professional performance (Minott, 2010).

Furthermore, it can be concluded that possessing a strong willingness and motivation to conduct, can support the purpose, specifically in cases wherein learning is self-directed. If a person aims to fulfill or learn something, and nobody is obliging them to do it, as is the example of self-directed learning, their opportunities of really performing it enhance, hence their goal is facilitated (Kyndt and Baert, 2013). When people were motivated, they had a greater degree of engagement in PD that can assist them with upgrading their information on a topic taking into consideration issues such as innovations, and sharing knowledge with other educators, professionals, and scholars. Furthermore, educators must become more and more prepared to grow, execute, and exchange practices, information, and values that meet learners’ demands to improve their teaching competencies (El-Hani and Greca, 2013). Utilizing a self-directed PD might motivate educators to reengage in their professional education (Colbert et al., 2008).

## Implications and Suggestions for Further Research

This review has implications for administrators since much of the funds for PD seem to be used for platforms that are not pleasing for educators and are not associated with their professional needs (Darling-Hammond et al., 2009; Wei et al., 2010). Nonetheless, through self-directed PD, educators are allowed to develop their PD plans, strengthening them to make decisions regarding the content of that plan is a highly different method of PD and has suggestions regarding how assets are utilized for PD and decision-making at the school level.

Self-directed PD motivates self-reflection, dedication, and accountability with high levels of motivational viewpoints and therefore, increasing self-consent. Self-directed PD leads to one gaining competence as well as obtaining a higher level of comprehension (Day and Lance, 2004). Self-directed PD is adopted by people who apprehend the necessity to grow and self-adjust their conduct as a result. Enhancing one’s skills through self-directed PD requires people first to be interested in and motivated to take part in tasks.

Educators’ PD chances can meet self-directed needs for educational evolution, which could enhance the motivation to continue attempting and solving boundaries. Based on the review of the literature, educator trainers and institution supervisors have to plan a few educational courses to get EFL educators familiar with practical and theoretical fundamentals of self-directed PD tactics and hold conferences to ask educators to use those tactics and enhance their reflectivity and offer feedback to them to provoke and increase their motivation and grit. Grit as a personal construct has also been proved to foresee educator presentation prospectively and resulted in educators’ greater influence at schools (Duckworth et al., 2009). Indeed, grit portrayed persistence in handling difficulties, and maintaining endeavors and interest throughout the years despite encountering setbacks, and flexibility, and hardships in attaining goals (Duckworth et al., 2007). Educators who can sustain a zeal for work, deal with intermittent difficulties, and not lose hope in the middle of the process demonstrated the persistence in endeavors at the center of the characterization of grit.

On the one hand, organizational education is generally planned to relay information and abilities to students and on the other hand, self-directed PD, as practiced by educators, altered their functions from inactive recipients to active students building their information through contemplation, cooperation, and connecting (Alshaikhi, 2018). It engaged educators actively in real assignments associated with their class practice involving teaching, evaluation, and monitoring (Timperley, 2011). It is proved that a component of the subjects’ education was societally debated and they talked of the extent to which their information and abilities were improved due to involvement, association, motivation, perseverance, and cooperation in the self-directed PD. By being actively engaged in a society of teachers exchanging and debating reciprocated worries, teacher educators were capable of developing their comprehension, building their self-esteem and motivation, and enhancing their practices. Teacher trainers should try to develop self-directed professional attentiveness among the teachers since self-directed PD programs are the prominent for the PD of teachers, to know about the prerequisite for development and the informed knowledge. Indeed, through these programs presented by teacher trainers in training programs, trainers can provide a range of facilities to assist teachers in making changes in their teaching strategies and styles and update their knowledge that consequently increasing their grit and motivation. Considering the nature of self-directed PD, future empirical studies may be carried out to search for other types of PD that might affect educators’ grit and motivation. More empirical inquiries can be conducted to collect not only quantitative but also qualitative data on the efficiency of self-directed concerning grit and motivation and even other concepts of positive psychology.

## Author Contributions

The author confirms being the sole contributor of this work and has approved it for publication.

## Funding

The work was supported by the practice of College English reform based on “output-oriented method” (SJGY20180545) and Cognitive description and evaluation of English language under two-way culture guidance (145109340).

## Conflict of Interest

The author declares that the research was conducted in the absence of any commercial or financial relationships that could be construed as a potential conflict of interest.

## Publisher’s Note

All claims expressed in this article are solely those of the authors and do not necessarily represent those of their affiliated organizations, or those of the publisher, the editors and the reviewers. Any product that may be evaluated in this article, or claim that may be made by its manufacturer, is not guaranteed or endorsed by the publisher.
